# Correlation and discriminatory performance of non-invasive serum fibrosis indices according to liver disease etiology using two-dimensional shear wave elastography as reference

**DOI:** 10.1016/j.clinsp.2026.101187

**Published:** 2026-10-01

**Authors:** María Elena Galaviz-Valdez, Liliana Torres-González, José Raúl Izaguirre-García, Linda E. Muñoz-Espinosa, Diana Patricia Moreno-Peña, Diana Raquel Rodríguez-Rodríguez, Idalia A. Cura-Esquivel, Paula Cordero-Pérez

**Affiliations:** a Universidad Autónoma de Nuevo León, Department of Internal Medicine, University Hospital Dr. José E. González, Monterrey, Nuevo León, Mexico; b Universidad Autónoma de Nuevo León. Liver Unit, Department of Internal Medicine, University Hospital Dr. José E. González, Monterrey, Nuevo León, Mexico; c Universidad Autónoma de Nuevo León, Department of Pediatrics, University Hospital Dr. José E. González, Monterrey, Nuevo León, Mexico

**Keywords:** Liver fibrosis, Non-invasive fibrosis assessment, Shear wave elastography, Viral hepatitis, Metabolic-associated steatotic liver disease, Alcohol-related liver disease

## Abstract

**Background/objectives:**

Non-invasive serum fibrosis indices are widely used for liver fibrosis assessment; however, their correlation with liver stiffness and diagnostic performance may vary according to liver disease etiology. This study aimed to evaluate the etiology-dependent correlation between multiple serum Non-Invasive Test (NITs) indices and liver stiffness measured by two-dimensional Shear Wave Elastography (2D-SWE), and to explore their ability to discriminate patients according to 2D-SWE-based fibrosis categories.

**Methods:**

We conducted a retrospective cross-sectional study including 105 patients with chronic liver disease. Serum NITs indices were correlated with liver stiffness measured by 2D-SWE using Pearson or Spearman correlation Exploratory ROC curve analyses were performed to assess the ability of serum indices to discriminate 2D-SWE-based fibrosis categories by etiology.

**Results:**

Strong intercorrelations were observed among multiple serum NITs indices across all etiological groups, indicating substantial collinearity. In metabolic-associated steatotic liver disease, alcohol-related liver disease, and metabolic dysfunction-associated alcohol-related liver disease, correlations between serum indices and liver stiffness were weak to moderate. In contrast, viral hepatitis showed moderate and statistically significant correlations between several indices and 2D-SWE, with more consistent diagnostic performance in ROC analyses. Autoimmune hepatitis demonstrated an intermediate pattern, with modest but significant associations between selected indices and liver stiffness. Exploratory ROC analyses showed heterogeneous discriminatory performance across etiologies.

**Conclusions:**

Serum NITs indices exhibit high collinearity and etiology-dependent performance. Their correlation with liver stiffness and their discriminatory performance against 2D-SWE-based fibrosis categories vary according to underlying liver disease supporting the complementary rather than interchangeable use of serum indices and elastography.

## Highlights

• 2D-SWE and serum indices show etiology-dependent correlation in liver fibrosis.
• Serum indices showed moderate correlation with 2D-SWE in MASLD.
• Correlations between serum indices and 2D-SWE were weak in ALD.
• Multiple serum indices correlated significantly with 2D-SWE in viral hepatitis.
• Etiological stratification is essential for non-invasive fibrosis staging.

## Introduction

Chronic Liver Diseases (CLD) represent a significant global health burden, with fibrosis serving as the common pathway toward cirrhosis and hepatocellular carcinoma.^1^ While liver biopsy remains the historical reference standard,^2^ its limitations have catalyzed the adoption of Non-Invasive Fibrosis Tests (NITs).^3^ Several serum NITs indices such as the Aspartate aminotransferase to Platelet Ratio Index (APRI), Fibrosis-4 Index (FIB-4), Göteborg University Cirrhosis Index (GUCI), King’s Score (KS), and Forns index (Forns) initially validated in Viral Hepatitis(VH),^4^, ^5^, ^6^, ^7^, ^8^ are now applied across etiologies including Metabolic-Associated Steatotic Liver Disease (MASLD), Alcohol-related Liver Disease (ALD), and Autoimmune Hepatitis (AIH), though performance varies by disease context.^9^, ^10^, ^11^, ^12^, ^13^, ^14^

Two-Dimensional Shear Wave Elastography (2D-SWE) offers a robust, real-time imaging alternative for fibrosis staging.^15^ However, limited elastography access maintains the clinical relevance of serum NITs indices as accessible diagnostic tools.^4^ Despite their widespread use, comparative data regarding the correlation between multiple serum NITs indices and 2D-SWE across diverse etiologies, particularly within Latin American cohorts, remain scarce.^16^, ^17^, ^18^, ^19^, ^20^

This study aimed to evaluate the etiology-dependent correlation between multiple serum NITs indices and liver stiffness measured by 2D-SWE, and to explore the discriminatory performance of these indices using 2D-SWE-based fibrosis categories across different liver disease etiologies.

## Material and methods

### Study design and setting

This was an observational, retrospective, cross-sectional study conducted at the Liver Unit including 105 patients with CLD of the “Dr. José Eleuterio González” University Hospital, Universidad Autónoma de Nuevo León, a tertiary referral center in northeastern Mexico (Fig. 1). The study analyzed data obtained from institutional medical records from January 2020 to September 2025 of patients with CLD who underwent liver stiffness assessment by 2D-SWE as part of routine clinical evaluation.

**Fig. 1:**
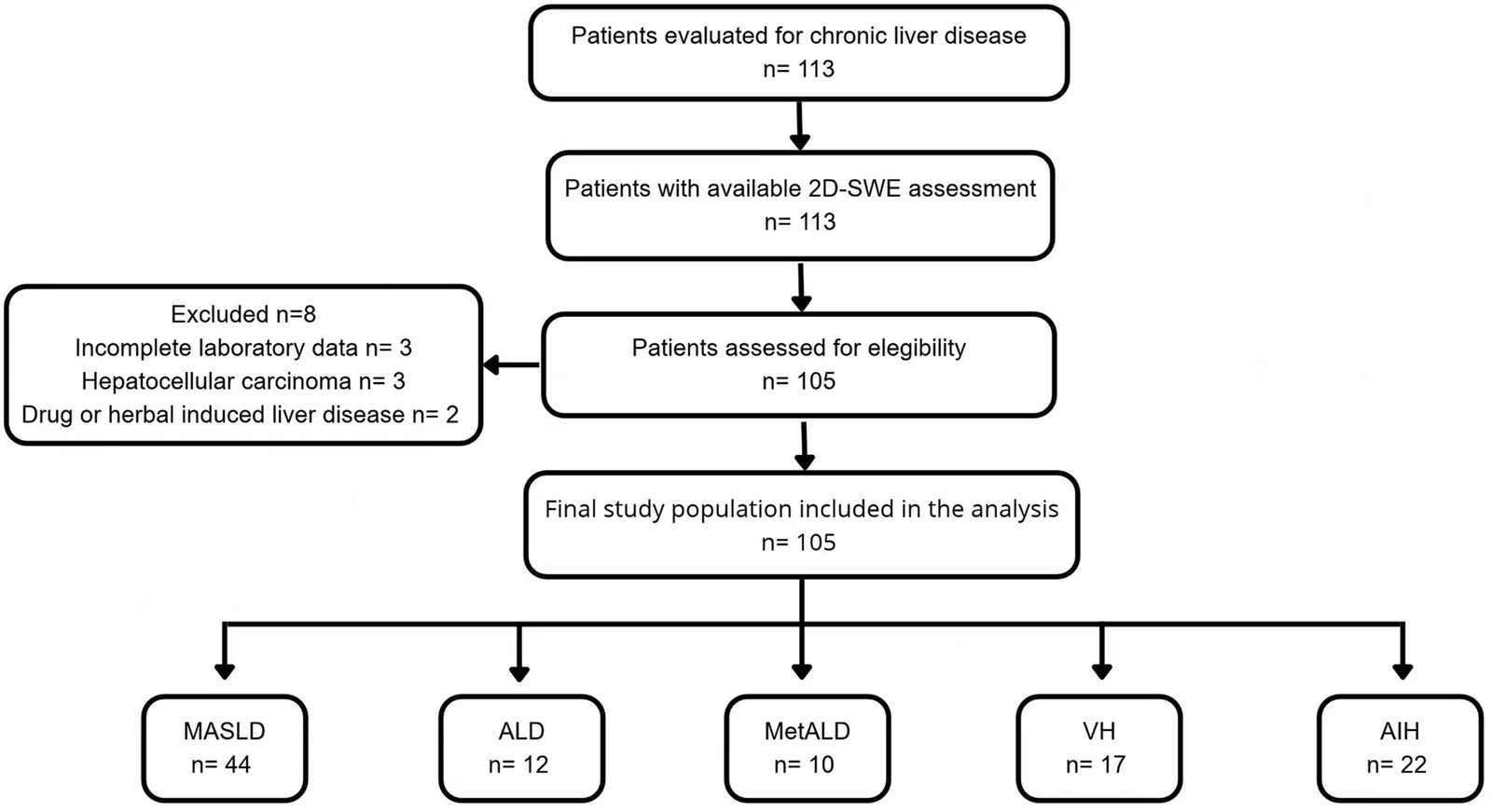
Flowchart of patient selection and study population. A total of 113 patients evaluated for chronic liver disease underwent two-Dimensional Shear Wave Elastography (2D-SWE). Eight patients were excluded due to incomplete laboratory data (*n* = 3), hepatocellular carcinoma at the time of evaluation (*n* = 3), or drug- or herbal-induced liver disease (*n* = 2). The final study population included 105 patients, who were classified according to liver disease etiology as metabolic dysfunction-associated steatotic liver disease (MASLD, *n* = 44), alcohol-related liver disease (ALD, *n* = 12), metabolic dysfunction-associated steatotic liver disease with alcohol consumption (MetALD, *n* = 10), viral hepatitis (VH, *n* = 17), and autoimmune hepatitis (AIH, *n* = 22).

### Study population

We consecutively included adult patients (≥18-years) with confirmed CLD (MASLD, ALD, Metabolic dysfunction-associated Alcohol-related Liver Disease (MetALD), VH, or AIH), diagnosed by standard clinical and biochemical criteria. Inclusion required a 2D-SWE examination and complete laboratory data within a 15-day interval. Exclusion criteria were incomplete records, lack of temporal concordance, presence of hepatocellular carcinoma, insufficient data for index calculation, drug- or herbal-induced liver disease, or pregnancy.

### Data collection

Demographic, clinical, biochemical, and hematological data were obtained from electronic institutional records. To ensure confidentiality, each patient was assigned a coded identifier, and no directly identifiable personal information was included in the database.

Collected variables included age, sex, etiology of liver disease, liver stiffness values measured by 2D-SWE, and laboratory parameters: total protein, globulin, Aspartate Aminotransferase (AST), Alanine Aminotransferase (ALT), Alkaline Phosphatase (ALP), Gamma-Glutamyl Transferase (GGT), albumin, total bilirubin, direct bilirubin, indirect bilirubin, Lactate Dehydrogenase (LDH), glucose, Blood Urea Nitrogen (BUN), creatinine, total cholesterol, erythrocytes, hemoglobin, platelet count, prothrombin time, and International Normalized Ratio (INR).

### Non-invasive fibrosis indices

All serum NITs indices analyzed in the present study and their corresponding formulas are shown in Table 1.

**Table 1:** Non-invasive serum-based fibrosis indices and their respective formulas.

Index	Formula	Reference
GPR	GGT / PLT	21
AAR	AST (IU/L) / ALT (IU/L)	22
Forns	7.811 − 3.131 × ln [PLT (10^9^/L)] + 0.781 × ln [GGT (IU/L)] + 3.467 × ln [age (years)] − 0.014 × cholesterol (mg/dL)	8
API	Age + PLT score	23
	Age: < 30 = 0, 30–39 = 1, 40–49 = 2, 50–59 = 3, 60–69 = 4, ≥ 70 = 5	
	PLT (10^9^/L): ≥ 225 = 0, 200–224 = 1, 175–199 = 2, 150–174 = 3, 125–149 = 4, < 125 = 5	
APRI	(AST/LSN) × 100/platelets(10^9^/L)	4
FIB-4	(age × AST [U/L]) / (PLT [10^9/L] × √ALT [U/L])	5
FQS	10 × [age (years) × AST (IU/L) × INR / [ALT (IU/L) × PLT (10^9^/L)];	24
FI	8.0 − 0.01 × PLT (10⁹/L) − albumin (g/dL)	25
^FCI^	[ALP (IU/L) × total bilirubin (mg/dL)] / [albumin (g/dL) × PLT (10^9^/L)]	26
^GUCI^	[(AST (IU/L) / ULN) × INR × 100 / PLT (10⁹/L)	6
CDS	Composite score based on PLT category + ALT/AST ratio category + INR category	27
	PLT (10^9^/L): ≥ 340 = 0, 280–339 = 1, 220–279 = 2, 160–219 = 3, 100–159 = 4, 40–99 = 5, < 40 = 6	
	ALT/AST: > 1.7 = 0, 1.2–1.7 = 1, 0.6–1.19 = 2, < 0.6 = 3	
	INR: < 1.1 = 0, 1.1–1.4 = 1, > 1.4 = 2	
Doha	8.5 − 0.2 × albumin (g/dL) + 0.01 × AST (IU/L) − 0.02 × PLT (10^9^/L)	28
KS	[age (years) × AST (IU/L) × INR] / PLT (10^9^/L)	7
S-index	1000 × GGT (IU/L) / [PLT (10^9^/L) × albumin^2^ (g/L^2^)	29

GPR, Gamma-Glutamyl transferase to Platelet Ratio; AAR, AST to ALT ratio; Forns, Forns index; API, Age-Platelet Index; APRI, AST to Platelet Ratio Index; FIB-4, Fibrosis-4 Index; FQS, Fibro-Q Score; FI, Fibrosis Index; FCI, Fibrosis-Cirrhosis Index; GUCI, Göteborg University Cirrhosis Index; CDS, Cirrhosis Discriminant Score; Doha, Doha score; KS, King’s Score; S-index, Serum fibrosis index, ULN, Upper Limit of Normal; PLT, Platelet Count.

### Liver stiffness measurement

Liver stiffness was assessed using 2D-SWE performed by trained personnel at the Liver Unit using an Aixplorer ultrasound system (Supersonic Imagine Aix-en-Provence, France). Liver stiffness values were recorded in kilopascals (kPa). A cutoff value of ≥ 8.0 kPa was used as a pragmatic 2D-SWE-based threshold for exploratory analyses across etiologies. This threshold was not intended to replace etiology-specific elastography interpretation or histological fibrosis staging. Because no universally accepted table of etiology-specific 2D-SWE cutoffs for significant fibrosis is available for all etiologies included in this cohort, ROC analyses based on this cutoff should be interpreted as exploratory. Examinations were conducted according to the manufacturer’s technical recommendations and international guidelines for liver elastography.

### Statistical analysis

Normality was assessed using the Kolmogorov-Smirnov test for the entire cohort and the Shapiro-Wilk test for etiological subgroups. Qualitative variables were expressed as frequencies, whereas quantitative variables were expressed as mean ± standard deviation for normally distributed data or as median and interquartile range for non-normally distributed data. Group comparisons were performed using ANOVA or the Kruskal-Wallis test, as appropriate, with post hoc analyses when indicated. Correlations between fibrosis indices and liver stiffness (2D-SWE) were evaluated using Pearson (parametric data) or Spearman (non-parametric data) coefficients. The discriminatory performance of serum fibrosis indices for identifying 2D-SWE-based fibrosis categories was explored using Receiver Operating Characteristic (ROC) curves and the Area Under the ROC Curve (AUC), stratified by etiology. AUC values were reported with 95% confidence intervals and corresponding *p*-values. A two-sided *p*-value < 0.05 was considered statistically significant. Analyses were conducted using GraphPad Prism v.10 (San Diego, CA, USA).

### Ethical considerations

The study protocol was reviewed and approved by the Research Ethics Committee and the Research Committee of the Faculty of Medicine and “Dr. José Eleuterio González” University Hospital, Universidad Autónoma de Nuevo León (record MI25-0010). The study was conducted in accordance with the principles of the Declaration of Helsinki and complied with national regulations for health research, including NOM-012-SSA3-2012 and in accordance with the Strengthening the Reporting of Observational Studies in Epidemiology (STROBE) Statement guidelines for cross-sectional studies. Given the retrospective nature of the study, informed consent was waived.

## Results

### Biochemical and hematological characteristics

The overall cohort had a mean age of 57±10 years, with 60% female patients. Age was comparable across etiological subgroups, ranging from 52±13 years in VH to 61±9 years in ALD. Female predominance was observed in AIH (86%), MASLD (73%), and VH (59%), whereas male patients predominated in ALD (92%) and MetALD (90%). Biochemical profiles (Table 2) showed preserved synthetic function across etiologies. However, ALD and MetALD exhibited significantly higher total bilirrubin, direct bilirubin and GGT levels compared with other etiologies. While transaminases overlapped, AST and ALT peaked in AIH and only ALT levels in ALD. Notably, platelet counts were significantly lower in ALD versus MetALD and VH. Renal function and other hematological parameters remained comparable across all diagnostic groups.

**Table 2:** Laboratory and hematological parameters among study groups.

Parameter	Overall group* (*n* = 105)	MASLD⁎⁎ (*n* = 44)	ALD⁎⁎ (*n* = 12)	MetALD⁎⁎ (*n* = 10)	VH⁎⁎ (*n* = 17)	AIH⁎⁎ (*n* = 22)
Total protein (g/dL)	7.2 ± 0.5	7.2 ± 0.5	7.3 ± 0.6	7.0 ± 0.6	7.2 ± 0.5	7.4 ± 0.6
Albumin (g/dL)	4.0 ± 0.5	4.2 ± 0.4	3.8 ± 0.5	3.6 ± 0.6	4.2 ± 0.4	3.9 ± 0.5
Globulin (g/dL)	3.2 ± 0.6	3.1 ± 0.5	3.4 ± 0.7	3.4 ± 0.7	2.9 ± 0.4	3.4 ± 0.7
Total bilirubin (mg/dL)	0.9 (0.6–1.7)	0.8 (0.5–1.2)	1.4 (0.9–2.2)	1.2 (0.6–2.8)	0.7 (0.5–1.5)	0.8 (0.6–2.3)
Direct bilirubin (mg/dL)	0.3 (0.2–0.5)	0.2 (0.2–0.4)	0.5 (0.2–0.7)	0.3 (0.2–1.2)	0.2 (0.2–0.4)	0.3 (0.2–1.3)
Indirect bilirubin (mg/dL)	0.6 (0.4–0.9)	0.5 (0.3–0.7)	0.9 (0.6–1.5)	0.8 (0.4–1.1)	0.5 (0.4–0.8)	0.5 (0.3–0.7)
AST (U/L)	40 (28–52)	35 (26–47)	42 (33–64)	38 (26–48)	35 (27–68)	49 (31–63)
ALT (U/L)	35 (25–52)	30 (25–48)	36 (27–67)	27 (25–48)	33 (19–59)	43 (28–68)
ALP (U/L)	105 (82–151)	111 (92–151)	116 (62–234)	116 (78–176)	94 ± 26	121 (80–226)
GGT (U/L)	43 (26–120)	47 (25–102)	61 (33–177)	111 (50–163)	35 (21–70)	43 (25–175)
LDH (U/L)	321 ± 78	325 ± 62	321 ± 72	303 ± 105	311 ± 65	329 ± 106
Glucose (mg/dL)	99 (91–110)	99 (94–110)	103 (90–108)	98 (87–110)	100 (87–112)	100 (90–104)
BUN (mg/dL)	14 (11–19)	15 (12–20)	13 (13–21)	12 (10–18)	14 (10–17)	15 (11–19)
Creatinine (mg/dL)	0.9 (0.7–1.0)	0.9 (0.8–1.1)	0.9 (0.7–1.1)	0.9 (0.7–1.1)	0.8 (0.7–0.9)	0.8 (0.7–0.9)
Cholesterol (mg/dL)	176 ± 53	181 ± 49	171 ± 41	137 ± 57	183 ± 31	180 ± 71
Erythrocytes (×10^6^/µL)	4.6 ± 0.9	4.5 ± 0.7	4.8 ± 0.5	4.4 ± 0.8	5.2 ± 1.4	4.5 ± 0.7
Hemoglobin (g/dL)	13.7 ± 2.2	13.6 ± 2.2	14.9 ± 1.0	13.2 ± 1.7	14.1 ± 3.2	13.6 ± 1.8
Platelet count (cells/µL)	186,129±87,524ᵃ^,^ᵇ	200,205±77,709	132,125±69,484	175,900±98,285	188,647±110,518	190,136±86,279
Prothrombin time (seg)	12.5 (11.4–13.9)	12.2 (11.4–13.5)	13.6 (11.3–14.4)	14.4 (12.8–16.1)	12.1 (11.7–13.3)	12.4 (11.1–14.5)
INR	1.21 ± 1.02	1.32 ± 1.57	1.17 ± 0.16	1.26 ± 0.17	1.11 ± 0.11	1.10 ± 0.14

Data are presented as mean ± standard deviation (parametric data) or median and interquartile range (non-parametric data), as appropriate. AST, Aspartate Aminotransferase; ALT, Alanine Aminotransferase; ALP, Alkaline Phosphatase; BUN, Blood Urea Nitrogen; GGT, Gamma-Glutamyl Transferase; INR, International Normalized Ratio; LDH, Lactate Dehydrogenase; U/L, Units per Liter; MASLD, Metabolic-Associated Steatotic Liver Disease; ALD, Alcohol-related Liver Disease; MetALD, Metabolic Dysfunction-Associated Alcohol-Related Liver Disease; AIH, Autoimmune Hepatitis; VH, Viral Hepatitis. *p* < 0.0001.

a ALD vs. MetALD.

b ALD vs. VH.

⁎ Kolmogorov-Smirov (Overall group).

⁎⁎ Shapiro Wilk (Subgroups etiologies).

### Comparison of non-invasive fibrosis indices and 2D-SWE among study groups

Most serum-based fibrosis indices (GPR, API, APRI, FIB-4, FQS, FI, FCI, GUCI, CDS, Doha, KS, and S-index) showed overlapping values across etiologies without statistical significance. However, AAR was higher in MetALD vs. MASLD (*p* = 0.0463), and Forns index was higher in ALD vs. MASLD (*p* = 0.0498). 2D-SWE liver stiffness differed significantly across groups, being higher in ALD (*p* = 0.0216) and MetALD (*p* = 0.0248) compared to MASLD. Conversely, stiffness values in VH and AIH were comparable to MASLD (Table 3).

**Table 3:** Comparison of non-invasive hepatic fibrosis indices and 2D-SWE among study groups.

Index	MASLD (*n* = 44)	ALD (*n* = 12)	MetALD (*n* = 10)	VH (*n* = 17)	AIH (*n* = 22)	p-value
GPR	0.5 (0.3–1.6)	1.7 (0.5–3.5)	1.4 (0.7–2.6)	0.4 (0.2–2.1)	0.9 (0.3–2.0)	NS
AAR	1.1 ± 0.4	1.2 ± 0.5	1.6 ± 0.7	1.1 ± 0.4	1.1 ± 0.4	a0.0463
Forns	6.1 ± 2.0	8.2 ± 2.2	7.7 ± 2.5	5.9 ± 2.6	6.2 ± 2.5	b0.0498
API	5.0 ± 2.2	6.7 ± 2.2	6.3 ± 2.2	5.2 ± 2.7	5.2 ± 3.0	NS
APRI	0.4 (0.3–0.8)	1.0 (0.5–2.3)	0.7 (0.3–1.1)	0.5 (0.3–1.8)	0.70 (0.4–1.3)	NS
FIB-4	1.7 (1.1–3.1)	3.6 (1.7–7.8)	3.3 (1.7–4.5)	1.7 (1.0–5.1)	2.0 (1.3–4.9)	NS
FQS	3.2 (2.2–6.2)	5.8 (3.6–18.4)	10.0 (3.3–12.5)	3.8(1.8–8.3)	4.2 (1.8–7.2)	NS
FI	1.6 ± 1.2	2.7 ± 1.1	2.6 ± 1.3	1.8 ± 1.4	2.1 ± 1.1	NS
FCI	0.1 (0.1–0.2)	0.5 (0.1–2.2)	0.2 (0.1–1.0)	0.08 (0.03–0.3)	0.2 (0.05–0.7)	NS
GUCI	0.4 (0.3–1.0)	1.3 (0.5–2.8)	0.9 (0.4–1.4)	0.5 (0.3–2.2)	0.7 (0.4–1.6)	NS
CDS	4.9 ± 1.8	6.5 ± 2.4	6.6 ± 2.4	5.3 ± 2.3	5.3 ± 2.3	NS
Doha	3.7 ± 2.3	5.5 ± 1.6	4.6 ± 2.1	4.4 ± 2.4	4.4 ± 1.9	NS
KS	9.2 (6.4–23.9)	29.4 (12.6–63.1)	24.7 (9.4–30.1)	8.3 (5.5–52.4)	16.2 (7.9–36.2)	NS
S-index	16.7 (7.1–44.9)	64.2 (10.7–178.1)	67.9 (18.8–115.4)	8.9 (4.3–58.7)	26.33 (7.1–60.9)	NS
2D-SWE (kPa)	7.7 (5.9–20.1)	29.3 (10.9–38.2)	32.2 (21.7–49.8)	7.4 (5.4–16.7)	12.3 (6.8–34.3)	b0.0216 / a0.0248

Data are presented as mean ± SD for parametric variables or median (IQR) for non-parametric variables. Intergroup comparisons were performed using ANOVA followed by Tukey’s post-hoc test, or Kruskal-Wallis followed by Dunn’s post-hoc test, as appropriate. *p* < 0.05 was considered statistically significant.

MASLD, Metabolic-Associated Steatotic Liver Disease; ALD, Alcohol-related Liver Disease; MetALD, Metabolic dysfunction-associated Alcohol-related Liver Disease; AIH, Autoimmune Hepatitis; GPR, Gamma-glutamyl transferase to Platelet Ratio; AAR, AST to ALT Ratio; Forns, Forns index; API, Age-Platelet Index; APRI, AST to Platelet Ratio Index; FIB-4, Fibrosis-4 index; FQS, Fibro-Q Score; FI, Fibrosis Index; FCI, Fibrosis-Cirrhosis Index; GUCI, Göteborg University Cirrhosis Index; CDS, Cirrhosis Discriminant Score; Doha, Doha score; KS, King’s Score; S-index, Serum fibrosis index. NS, Not Significant.

a MASLD vs. MetALD.

b MASLD vs. ALD.

### Correlation between non-invasive serum liver fibrosis indices and 2D-SWE by etiology

Correlation heatmaps (Fig. 2, Fig. 3) revealed substantial collinearity among non-invasive fibrosis indices and liver stiffness measured by 2D-SWE according to liver disease etiology. In MASLD (Fig. 2A), strong parametric correlations occurred between Forns, API, FI, CDS, and Doha (*r* = 0.68–0.95; *p* < 0.001), while non-parametric analysis (Fig. 2B) showed high intercorrelations between APRI, FIB-4, FQS, GUCI, and KS (*r* = 0.83; *p* < 0.001). Overall, associations between several serum indices and 2D-SWE were moderate, with correlation coefficients ranging from 0.49 to 0.59 (*p* < 0.001).

**Fig. 2:**
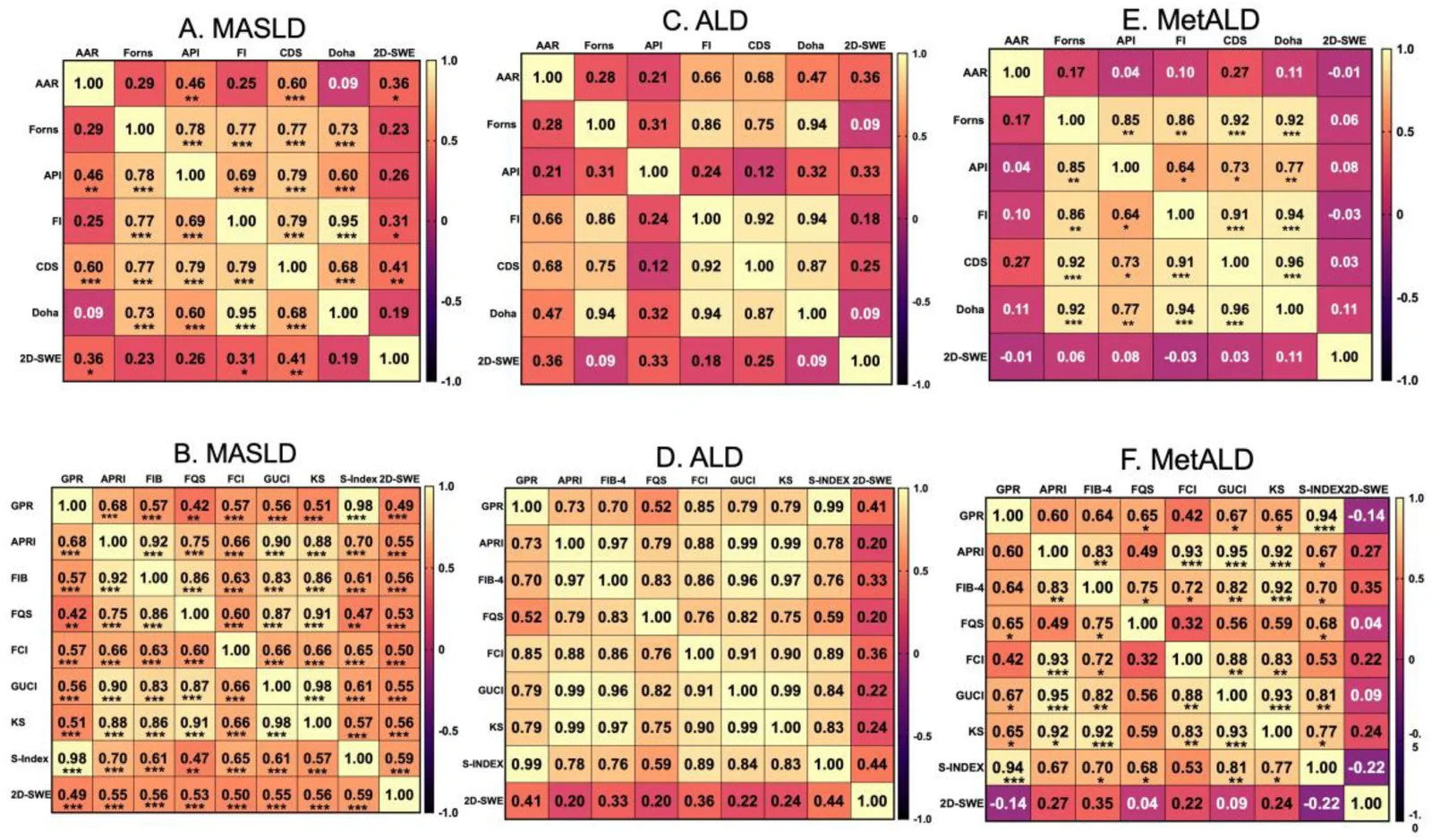
Correlation matrix by study group showing the associations between non-invasive serum fibrosis indices and liver stiffness measured by two-Dimensional Shear Wave Elastography (2D-SWE). (A, C, E) Parametric analysis using Pearson correlation; (B, D, F) Non-parametric analysis using Spearman correlation. Asterisks indicate the level of statistical significance in the correlation analysis: *p* < 0.05 (*), *p* < 0.01 (**), and *p* < 0.001 (***). Associations without asterisks did not reach statistical significance.

**Fig. 3:**
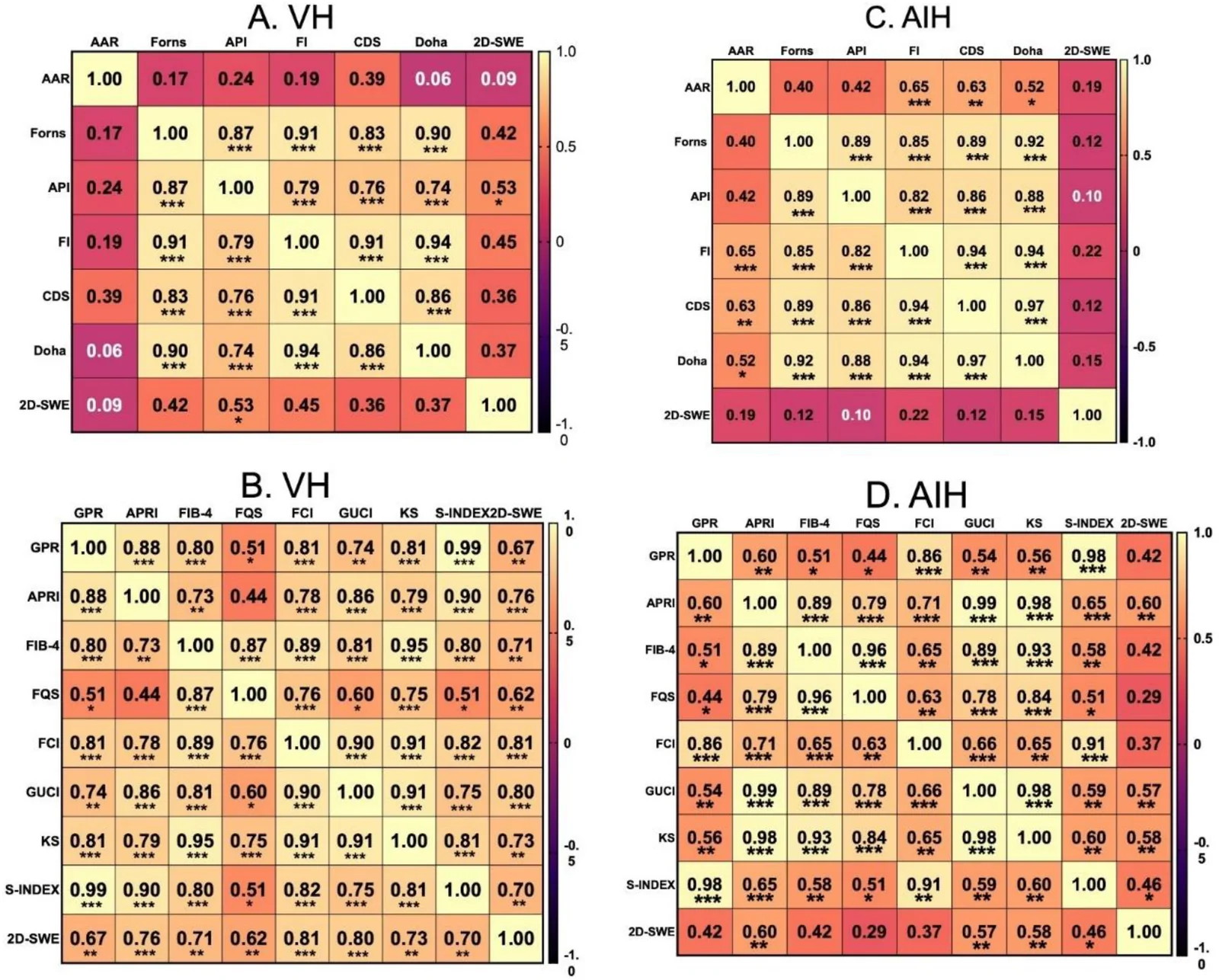
Correlation matrix by study group showing the associations between non-invasive serum fibrosis indices and liver stiffness measured by two-Dimensional Shear Wave Elastography (2D-SWE). (A, C) Parametric analysis using Pearson correlation; (B, D) Non-parametric analysis using Spearman correlation. Asterisks indicate the level of statistical significance in the correlation analysis: *p* < 0.05 (*), *p* < 0.01 (**), and *p* < 0.001 (***). Associations without asterisks did not reach statistical significance.

In ALD (Fig. 2C) and MetALD (Fig. 2E), similar strong collinearity was observed among serum indices Forns, FI, CDS, and Doha (*r* = 0.75‒0.94, *p* < 0.01 and *r* = 0.85‒0.96, *p* < 0.01, respectively); while non-parametric analysis in ALD (Fig. 2D) and MetALD (Fig. 2F) strong intercorrelations were identified among APRI, FIB-4, FQS, FCI, GUCI, KS, and S-index (*r* = 0.75, *p* < 0.01; *r* = 0.85‒0.96, *p* < 0.01, respectively). Associations between serum fibrosis indices and 2D-SWE were weak to moderated and did not reach statistical significance in both etiologies. Overall, the heat map analysis revealed substantial collinearity limited correlation with liver stiffness measured by 2D-SWE in patients with ALD and MetALD.

In the parametric analysis of VH (Fig. 3A) and AIH (Fig. 3C) serum indices maintained high collinearity between Forns, API, FI, CDS, and Doha (*r* = 0.74‒0.94, *p* < 0.01) and Forns, API, FI, CDS, and Doha (*r* = 0.82‒0.97, *p* < 0.001), respectively; while non-parametric analysis both subgroups showed for strong intercorrelations were identified among APRI, FIB-4, FQS, FCI, GUCI, KS, and S-index with *r* = 0.73, *p* < 0.01 for VH (Fig. 3B) and *r* = 0.63, *p* < 0.01 for AIH (Fig. 3D). In contrast to other etiological groups in VH, moderate and statistically significant correlations were observed between multiple serum-based indices and 2D-SWE (*r* = 0.62‒0.81, *p* < 0.01). Similarly, in AIH, moderate correlations were observed between 2D-SWE and indices such as APRI, GUCI, KS, and S-index (*r* = 0.46‒0.60, *p* < 0.05). Overall, the heat map analysis demonstrated substantial collinearity among non-invasive fibrosis indices and stronger or modest associations with liver stiffness measured by 2D-SWE in the VH and AIH, respectively.

### Exploratory ROC analysis of serum fibrosis indices for 2D-SWE-based fibrosis categories by disease etiology

ROC analyses (Figs. 4A‒C and 5A‒B) demonstrated heterogeneous discriminatory performance across etiologies. The corresponding AUC values, 95% confidence intervals, and *p*-values for each fibrosis index are summarized in Table 4.

**Fig. 4:**
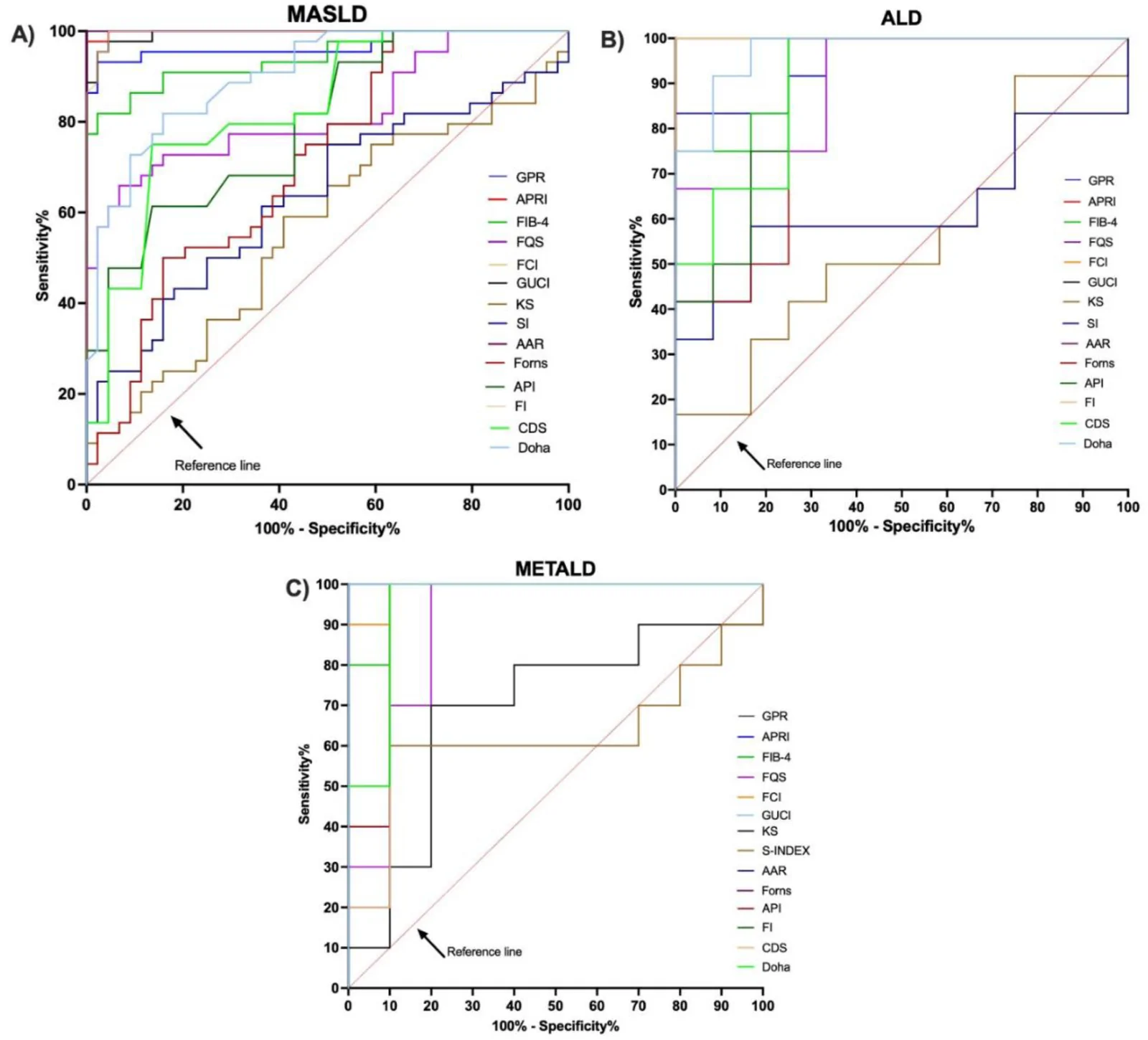
Receiver Operating Characteristic (ROC) curves assessing the discriminatory performance of non-invasive serum fibrosis indices across different liver disease etiologies, including (A) MASLD, (B) ALD and (C) MetALD. Liver fibrosis was assessed using two-dimensional shear wave elastography (2D-SWE), with a cutoff value ≥8 kPa indicating the presence of fibrosis. The dashed diagonal line represents the expected diagnostic performance by chance. MASLD, Metabolic Dysfunction-Associated Steatotic Liver Disease; MetALD, Metabolic Dysfunction-Associated steatotic Liver Disease with alcohol consumption; ALD, Alcohol-related Liver Disease.

**Fig. 5:**
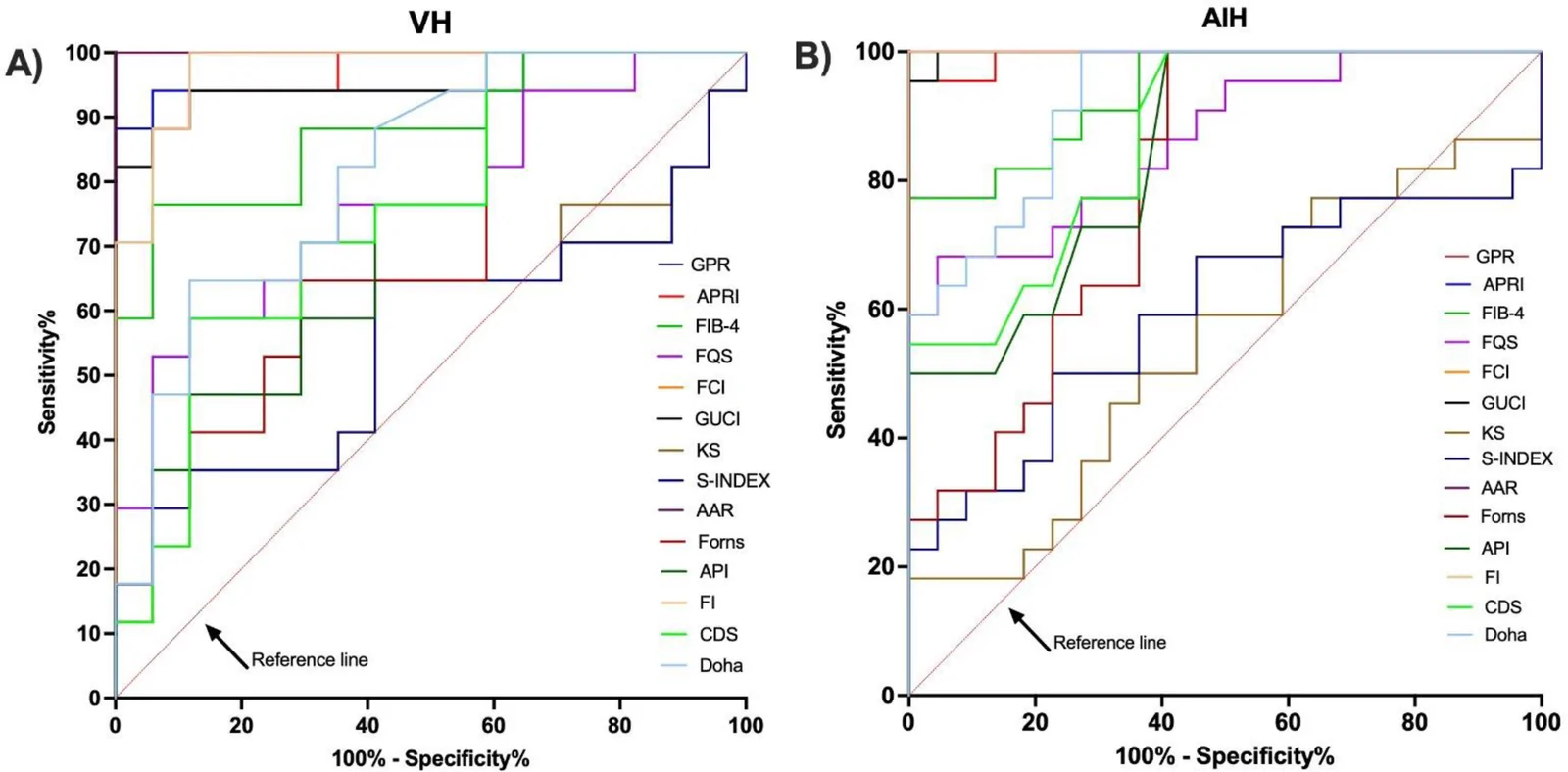
Receiver Operating Characteristic (ROC) curves assessing the discriminatory performance of non-invasive serum fibrosis indices across different liver disease etiologies, including (A) VH and (B) AIH. Liver fibrosis was assessed using two-Dimensional Shear Wave Elastography (2D-SWE), with a cutoff value ≥8 kPa indicating the presence of fibrosis. The dashed diagonal line represents the expected diagnostic performance by chance. VH, Viral Hepatitis; AIH, Autoimmune Hepatitis.

**Table 4:** Area under the receiver operating characteristic curve and discriminatory performance of non-invasive indices in across different chronic liver disease etiologies.

NITs	AUC (95% CI)	Sensitivity (%)	Specificity (%)	*p*
MASLD				
GPR	0.96 (0.93–1.00)	93.18	97.73	<0.0001
APRI	1.00 (0.99–1.00)	97.73	97.73	<0.0001
FIB-4	0.94 (0.89–0.99)	81.82	95.45	<0.0001
FQS	0.82 (0.73–0.91)	90.91	36.36	<0.0001
FCI	0.95 (0.92–1.00)	93.18	97.73	<0.0001
GUCI	0.98 (0.98–1.00)	95.45	97.73	<0.0001
KS	0.56 (0.44–0.68)	95.45	2.27	0.3166
S-Index	0.63 (0.51–0.75)	84.09	20.45	0.0276
AAR	1.00 (0.98–1.00)	95.45	97.73	<0.0001
Forns	0.71 (0.60–0.81)	90.91	38.64	0.0006
API	0.79 (0.70–0.88)	93.18	47.73	<0.0001
FI	0.98 (0.98–1.00)	97.73	95.45	<0.0001
CDS	0.82 (0.74–0.91)	97.73	47.73	<0.0001
Doha	0.90 (0.84–0.96)	81.82	84.09	<0.0001
MetALD				
GPR	1.00 (1.00–1.00)	100.00	100.00	0.0002
APRI	1.00 (1.00–1.00)	100.00	100.00	0.0002
FIB-4	0.98 (0.92–1.00)	100.00	90.00	0.0003
FQS	0.90 (0.75–1.00)	100.00	80.00	0.0025
FCI	0.99 (0.95–1.00)	100.00	90.00	0.0002
GUCI	1.00 (1.00–1.00)	100.00	100.00	0.0002
KS	0.69 (0.69–0.93)	80.00	60.00	0.1509
S-Index	0.65 (0.38–0.91)	70.00	30.00	0.2568
AAR	1.00 (1.00–1.00)	100.00	100.00	0.0002
Forns	0.92 (0.76–1.00)	90.00	90.00	0.0015
API	0.94 (0.82–1.00)	100.00	90.00	0.0009
FI	1.00 (1.00–1.00)	100.00	100.00	0.0002
CDS	0.92 (0.76–1.00)	100.00	90.00	0.0015
Doha	0.95 (0.84–1.00)	100.00	90.00	0.0007
ALD				
GPR	0.95 (0.87–1.00)	100.00	66.70	0.0002
APRI	1.00 (1.00–1.00)	100.00	91.67	<0.0001
FIB-4	0.94 (0.86–1.00)	100.00	75.00	0.0002
FQS	0.89 (0.77–1.00)	100.00	66.67	0.001
FCI	1.00 (1.00–1.00)	100.00	100.00	<0.0001
GUCI	1.00 (1.00–1.00)	100.00	100.00	<0.0001
KS	0.54 (0.58–0.78)	91.67	25.00	0.6861
S-Index	0.61 (0.37–0.86)	83.33	25.00	0.3263
AAR	1.00 (1.00–1.00)	100.00	100.00	<0.0001
Forns	0.86 (0.70–1.00)	100.00	75.00	0.0027
API	0.88 (0.75–1.00)	100.00	75.00	0.0012
FI	1.00 (1.00–1.00)	100.00	100.00	<0.0001
CDS	0.90 (0.78–1.00)	100.00	75.00	0.0008
Doha	0.97 (0.91–1.00)	100.00	83.33	<0.0001
VH				
GPR	0.96 (0.88–1.00)	94.12	94.12	<0.0001
APRI	0.97 (0.92–1.00)	94.12	88.24	<0.0001
FIB-4	0.88 (0.77–1.00)	76.47	94.12	0.0001
FQS	0.77 (0.61–0.93)	76.47	64.71	0.0076
FCI	1.00 (1.00–1.00)	100.00	100.00	<0.0001
GUCI	0.96 (0.88–1.00)	94.12	88.24	<0.0001
KS	0.56 (0.36–0.76)	64.71	58.82	0.5240
S-Index	0.55 (0.35–0.76)	64.71	58.82	0.5934
AAR	1.00 (1.00–1.00)	100.00	100.00	<0.0001
Forns	0.70 (0.52–0.88)	64.71	70.59	0.0439
API	0.73 (0.56–0.90)	76.47	58.82	0.022
FI	0.98 (0.93–1.00)	100.00	88.24	<0.0001
CDS	0.75 (0.59–0.92)	76.47	58.82	0.0114
Doha	0.82 (0.67–0.96)	88.24	58.82	0.0016
AIH				
GPR	0.99 (0.98–1.00)	100.00	86.36	<0.0001
APRI	1.00 (1.00–1.00)	100.00	100.00	<0.0001
FIB-4	0.94 (0.87–1.00)	100.00	63.64	<0.0001
FQS	0.86 (0.76–0.97)	86.36	59.09	<0.0001
FCI	1.00 (1.00–1.00)	100.00	100.00	<0.0001
GUCI	1.00 (0.99–1.00)	100.00	95.45	<0.0001
KS	0.55 (0.38–0.72)	59.09	54.55	0.5732
S-Index	0.60 (0.42–0.77)	68.18	54.55	0.2699
AAR	1.00 (1.00–1.00)	100.00	100.00	<0.0001
Forns	0.80 (0.66–0.93)	86.36	59.09	0.0008
API	0.85 (0.73–0.96)	100.00	59.09	<0.0001
FI	1.00 (1.00–1.00)	100.00	100.00	<0.0001
CDS	0.87 (0.76–0.97)	77.27	72.73	<0.0001
Doha	0.92 (0.85–1.00)	100.00	72.73	<0.0001

GPR, Gamma-Glutamyl transferase to Platelet Ratio; AAR, AST to ALT Ratio; Forns, Forns index; API, Age-Platelet Index; APRI, AST to Platelet Ratio Index; FIB-4, Fibrosis-4 Index; FQS, Fibro-Q Score; FI, Fibrosis Index; FCI, Fibrosis-Cirrhosis Index; GUCI, Göteborg University Cirrhosis Index; CDS, Cirrhosis Discriminant Score; Doha, Doha score; KS, King’s Score; S-index, Serum fibrosis index; AUC, Area Under the receiver operating characteristic Curve; MASLD, Metabolic-Associated Steatotic Liver Disease; ALD, Alcohol-related Liver Disease; MetALD, Metabolic dysfunction-associated Alcohol-related Liver Disease; VH, Viral Hepatitis; AIH, Autoimmune Hepatitis.

In MASLD, APRI and AAR showed the highest AUC values among the evaluated indices, followed by GUCI, FI, GPR, FCI, FIB-4 and Doha. In contrast, KS and S-index exhibited the lowest AUC values and did not demonstrate statistically significant discriminatory performance (Fig. 4A).

In ALD, APRI, FCI, GUCI, AAR and FI, yielded the highest AUC values, whereas KS and S-index showed the lowest AUC estimates, indicating limited discriminatory performance (Fig. 4B). However, the limited sample size in this subgroup warrants cautious interpretation of these results.

In MetALD, GPR, APRI, GUCI, AAR and FI showed the highest AUC values whereas KS and S-index showed the lowest AUC (Fig. 4C). However, because this subgroup included a limited number of patients, these estimates should be interpreted cautiously.

In VH, FCI, AAR, FI, APRI, GPR, GUCI demonstrated the highest AUC values, whereas KS and S-index showed the lowest AUC (Fig. 5A).

In AIH, APRI, FCI, GUCI, AAR, FI, Doha, GPR and FIB-4 showed the highest AUC values. Conversely, KS and the S-index showed limited diagnostic performance and did not reach statistical significance (Fig. 5B).

## Discussion

We evaluated the etiology-dependent correlation patterns between multiple non-invasive serum fibrosis indices and liver stiffness measured by 2D-SWE. We also explored the discriminatory performance of these indices for 2D-SWE-based fibrosis categories, recognizing that 2D-SWE is a non-invasive reference and not a histological gold standard.

In this study, ALD and MetALD presented the highest levels of AST, ALT, GGT, and bilirubin, consistent with a more inflammatory and cholestatic biochemical profile described for these etiologies.^30^ Notably, the particularly elevated GGT levels observed in MetALD support its role as a sensitive marker of oxidative stress and hepatocellular injury described for this etiology.^31^ The preservation of albumin and coagulation parameters across groups suggests a cohort predominantly in early fibrosis stages, as these synthetic markers typically decline only in advanced cirrhosis.^32^ Furthermore, the thrombocytopenia observed in alcohol-related groups likely reflects direct alcohol toxicity, hypersplenism, or bone marrow suppression.^32^ These findings support the classification of MetALD as a distinct clinical entity where overlapping metabolic and alcohol-induced mechanisms intensify biochemical alterations.^31^

In MASLD, we observed heterogeneous correlations between serum NITs indices and 2D-SWE. GPR showed a significant association, echoing emerging evidence of its utility in fatty liver contexts.^33^ Conversely, AAR and Forns demonstrated inconsistent or weak correlations, aligning with prior reports questioning their accuracy in MASLD.^34^ Notably, indices originally developed for VH, including API, FQS, FI, FCI, GUCI, CDS, KS, Doha, and S-index, showed moderate-to-high correlations, suggesting potential complementary value. However, APRI and FIB-4 showed higher discriminatory performance against 2D-SWE-based categories in this cohort; consistent with previous Mexican cohorts where FIB-4 outperformed APRI,^35^^,^^36^ however, these findings should be interpreted as exploratory because 2D-SWE was used as a non-invasive reference rather than histology. The high accuracy observed here compared to other studies^37^ may reflect specific population characteristics or a higher prevalence of significant fibrosis in our sample.

In contrast, ALD exhibited marked variability and overall weak correlations between serum NITs indices and 2D-SWE. Parameters such as GPR, AAR, Forns and API demonstrated limited associations, likely due to alcohol-induced biochemical fluctuations that confound traditional fibrosis scores.^38^^,^^39^ Even established tools like FIB-4 showed attenuated performance despite their inclusion in current guidelines.^4^ In MetALD, the combined metabolic and alcoholic variability appears to synergistically limit traditional index performance.^31^^,^^40^ Higher AUC values were observed in our study compared to the other etiological groups; however, these findings should be interpreted cautiously due to the limited number of patients in this subgroup.

In VH demonstrated the most consistent associations, with APRI and FIB-4 showing strong correlations with 2D-SWE, reinforcing their extensive validation.^7^^,^^21^ GPR, APRI, FCI, GUCI, AAR and FI, also showed higher AUC values (0.96‒1.00) consistent with previous FibroScan-based studies for some of these NITs.^7^^,^^21^ In AIH, evidence remains scarce^19^; however, our data showed modest-to-moderate correlations for APRI and FIB-4. Our findings highlight the importance of etiological stratification when selecting non-invasive fibrosis tools. The high degree of collinearity among indices suggests limited incremental benefit from combining multiple scores derived from similar laboratory parameters. The main limitation of this study is the absence of liver biopsy as the histological reference standard. Therefore, our findings should not be interpreted as true diagnostic accuracy for biopsy-defined fibrosis stages. Instead, they reflect the correlation and discriminatory performance of serum fibrosis indices against liver stiffness and fibrosis categories defined by 2D-SWE, which is itself a non-invasive method and may be influenced by inflammatory activity, cholestasis, hepatic congestion, and technical variability. Another limitation is the use of a single 2D-SWE cutoff across etiologies. Although this approach allowed a uniform exploratory comparison across the cohort, liver stiffness thresholds may differ according to disease etiology, elastography technique, ultrasound system, and clinical context. In addition, the small sample sizes in the ALD and MetALD subgroups limit the stability of ROC estimates; therefore, these analyses should be interpreted as exploratory.

## Conclusions

In this study, serum NITs indices exhibit substantial inter-index collinearity across all etiologies, reflecting shared biochemical components rather than independent measures of fibrosis. Their correlation with 2D-SWE and their discriminatory performance for 2D-SWE-based fibrosis categories were etiology-dependent, with stronger associations observed in VH and AIH and more limited or variable associations in MASLD, ALD, and MetALD. Consequently, clinicians should prioritize etiology-specific stratification when interpreting these tools. These findings support the complementary, rather than interchangeable, use of serum indices and elastography in the non-invasive assessment of liver fibrosis. Further studies using histological or longitudinal clinical outcomes as reference standards are needed to validate these findings.

## Declaration of generative AI in scientific writing

During the preparation of this work, the authors used basic AI tools to check grammar, spelling, and references.

## Authors’ contributions

María Elena Galaviz-Valdez, Liliana Torres-González, Paula Cordero-Pérez: Conceptualization; Data curation; Investigation; Methodology; Project administration; Resources; Supervision; Visualization; Formal analysis; Writing-original draft; Preparation; Writing-review and editing. José Rául Izaguirre-García, Linda E. Muñoz-Espinosa: Investigation; Methodology; Investigation; Formal analysis; Writing-review and editing. Diana Patricia Moreno-Peña, Diana Raquel Rodriguez-Rodriguez, Idalia A. Cura-Esquivel: Investigation; Formal analysis; Writing-review and editing.

## Funding

This research did not receive any specific grant from funding agencies in the public, commercial, or not-for-profit sectors.

## Conflicts of interest

The authors declare that they have no known competing financial interests or personal relationships that could have appeared to influence the work reported in this paper.

## Data Availability

Data available from the corresponding author upon reasonable request.
